# Implementation of a Low-Cost Data Acquisition System on an E-Scooter for Micromobility Research

**DOI:** 10.3390/s22218215

**Published:** 2022-10-26

**Authors:** Ana María Pérez-Zuriaga, David Llopis-Castelló, Víctor Just-Martínez, Alejandra Sofía Fonseca-Cabrera, Carlos Alonso-Troyano, Alfredo García

**Affiliations:** Highway Engineering Research Group, Universitat Politècnica de València, 46022 Valencia, Spain

**Keywords:** instrumented e-scooter, micromobility safety, sensors, Raspberry Pi, data acquisition system

## Abstract

In recent years, cities are experiencing changes in the ways of moving around, increasing the use of micromobility vehicles. Bicycles are the most widespread transport mode and, therefore, cyclists’ behaviour, safety, and comfort have been widely studied. However, the use of other personal mobility vehicles is increasing, especially e-scooters, and related studies are scarce. This paper proposes a low-cost open-source data acquisition system to be installed on an e-scooter. This system is based on Raspberry Pi and allows collecting speed, acceleration, and position of the e-scooter, the lateral clearance during meeting and overtaking manoeuvres, and the vibrations experienced by the micromobility users when riding on a bike lane. The system has been evaluated and tested on a bike lane segment to ensure the accuracy and reliability of the collected data. As a result, the use of the proposed system allows highway engineers and urban mobility planners to analyse the behaviour, safety, and comfort of the users of e-scooters. Additionally, the system can be easily adapted to another micromobility vehicle and used to assess pavement condition and micromobility users’ riding comfort on a cycling network when the budget is limited.

## 1. Introduction

Mobility in cities is changing worldwide, increasing the so-called micromobility. This concept of mobility includes all transportation modes that allow their users to make a hybrid usage and behave either as a pedestrian or as a vehicle at their convenience or when necessary [1]. Bicycle riding is the most widespread micromobility transport mode, followed by stand-up electric scooters (e-scooters) [2]. Understanding their users’ behaviour, evaluating their safety, and monitoring and assessing the conditions of the facilities they use is key to ensuring an effective impact on urban mobility.

Several studies have focused on the analysis of cyclists’ behaviour and their interaction with motorized vehicles. The main variables analysed are the speed and acceleration of the bicycle, and speed of the motorized vehicle passing a bicycle and the lateral clearance it leaves during the manoeuvre. Most studies based their research on data collected thanks to an instrumented bicycle [3]. Used instrumented bicycles are usually equipped with expensive laser systems to collect distance and speed data, a global positioning system (GPS) to collect position and speed data, and video cameras [4]. In order to reduce costs, several studies have based their findings on custom-made devices using: (i) distance sensors connected to an Arduino Uno microcontroller card [5]; (ii) an ultrasonic sensor, a GPS receiver, (iii) a microcontroller and a data logger [6]; (iv) a device including an Adafruit microprocessor, GPS sensor, ultrasonic sensor, and lithium ion [7]; (v) a device based on Raspberry Pi, with a micro lidar as a distance sensor, a GPS, and a camera [8].

Instrumented bicycles are also used to analyse cycling safety based on the recognition of hard breaking [9]. They monitored the dynamic of an instrumented bicycle with cameras, accelerometers, and two GPS systems working at different acquisition frequencies. The results of this study confirmed that at least a GPS signal with a sampling frequency of 1 Hz is needed to track the rider’s behaviour to identify events.

Cycling safety has also been studied using GPS cycling trip data from a smartphone application to extract deceleration rates at intersections and along segments [10]. They started as a technological limitation in the frequency of the smartphone GPS data which cannot be sufficient for extracting reliable bicycle deceleration data.

Moreover, some studies intended to use an instrumented bicycle for assessing the riding quality of cycling facilities based on vibration measurements. In order to register this variable, Bil et al. [11] used a system with a GPS and a data logger with accelerometers (MSR145s); Chou et al. [12] equipped a bicycle with an accelerometer, a distance measurement instrument, a speed meter, and a data acquisition system; and Ambroz et al. [13] used a system based on Raspberry Pi with two tri-axial accelerometers, a wire potentiometer, and a magnetic reed-switch based speed sensor. In addition to these sensors, vertical acceleration measurements for assessing the quality of bicycle infrastructures have also been collected using a smartphone [14,15].

Although cyclists’ behaviour, safety, and comfort have been widely researched, there are fewer studies on e-scooters. The interactions between e-scooter riding and the environment settings were studied based on data recorded by a mobile sensing system [16]. This system collected data for quantifying surrogate safety metrics in terms of vibrations, speed changes, and proximity to surrounding objects. It includes the following sensing systems: (i) a GPS; (ii) an inertial measurement unit (IMU) combining a three-axis gyroscope and three-axis accelerometer; (iii) a LIDAR Scanner; and (iv) a video camera. All sensors were synchronized with a Raspberry Pi platform for data acquisition, processing, and storing. They were powered by a portable low-voltage battery pack.

Vibrations have also been the base for monitoring and assessment urban road pavement studies. Smartphone sensors have been used to collect data for the assessment of pavement conditions and definition of key performance indicators (KPI) of e-scooter users’ ride comfort and safety [17,18]. The variables collected by the android application were acceleration and position data. The analysis of this data also allowed the identification of high-severity pavement distresses.

E-scooter riding comfort has been also analysed using an Arduino-based data acquisition system at a sampling frequency of 200 Hz, allowing the collection of acceleration and speed measurements [19]. The system was based on the Nano 3.x microcontroller from Arduino, which converted the signal from the accelerometers and AH49E Hall-effect linear magnetic detector through analogue–digital converters into integer values that were stored in text files on an SD card.

In this context, the aim of this research is the development of a low-cost, open-source data acquisition system (DAS) to be assembled on an e-scooter for the study of micromobility users’ behaviour, safety, and riding comfort. This DAS is based on a Raspberry Pi platform for data acquisition, processing, and storing, and includes: (i) an IMU; (ii) two ultrasonic sensors; (iii) an external battery. This system is complemented by a video camera equipped with a GPS.

As a result, the system allows measuring the speed and accelerations of the e-scooter, the georeferenced position, the lateral clearance during overtaking and meeting manoeuvres, and the speed of the micromobility vehicles interacting with the e-scooter. Moreover, the instrumented e-scooter allows measuring the vibrations experienced by micromobility users when riding along cycling facilities.

The rest of the paper is organized as follows: Section 2 presents the components and software used to design the instrumented e-scooter; Section 3 describes a case study focusing on the application of the instrumented e-scooter to check the accuracy and reliability of the data collected; Section 4 shows a discussion of the findings of the study, including the limitations of the proposed data acquisition system; and Section 5 includes the main conclusions.

## 2. Materials and Methods

**Figure 1:**
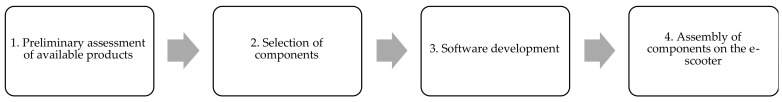
Stages for the e-scooter instrumentation.

This section presents the components that are part of the low-cost open-source data acquisition system (DAS) and describes the assembly of these components on the e-scooter. Figure 1 summarizes the stages of the instrumentation of the e-scooter. The first step was the analysis of the devices available on the market. As a result, the devices to be used in the instrumentation were selected. Subsequently, several scripts were programmed in Python version 3.7 to operate the devices. Finally, the devices were assembled on the e-scooter and then tested to ensure reliable data collection.

### 2.1. Preliminary Assessment

The first step was to conduct a preliminary study of the products available on the market and possible technical solutions. These solutions must be capable of recording the necessary data to characterize the following variables:

- E-scooter position
- E-scooter speed and accelerations
- Lateral clearance left during overtaking and meeting manoeuvres
- Speed developed by the micromobility user meeting or overtaking the e-scooter

In previous research [4], bicycles were instrumented to characterize overtaking manoeuvres, measuring bicycle speed, lateral clearance left by the motorized vehicle when overtaking a bicycle, and overtaking speed. These bicycles were equipped with a Laser Technology Inc. T200/T100 laser system, that consist of a couple of laser rangefingers, perpendicular to bicycle axis, on the front and the rear of the bicycle. These devices allow measuring lateral clearance and relative speed.

Bicycles also included two high-definition video cameras (Garmin VIRB Elite) with a 10 Hz GPS. Bicycle position and speed are obtained by the video camera whereas motorized vehicle speed is calculated considering the relative speed measured by the T200/T100 laser system and the bicycle speed.

In order to instrument the e-scooter, the first option was to use the available devices. However, there was a major drawback: the size and weight of the T200/T100 laser system and the required battery. This is not a special restriction for a bicycle, but it is for an e-scooter since its stability is lower.

Therefore, considering the inability of using the available devices and the cost of smaller devices, the next option was to develop a custom-made data acquisition system. Previous studies have used for data management either microcontrollers (e.g., Arduino) or single board nano computers (e.g., Raspberry Pi), and 3D printers and laser cut for personalized cases.

For this study, a data acquisition system based on the Raspberry Pi 4B was developed due to the excellent online support, high-level connectivity to multiple devices, and its small size and low price (~70 €). An aluminium case was used as a lightweight protective case for the sensors.

Raspberry Pi technology is a relatively new system that has been developed by the Raspberry Pi Foundation in the United Kingdom. Specifically, it is a reduced board or single board computer, low cost, and with high potential in the development of different types of engineering projects, as it can be adapted to many different types of situations. In addition to the board, the following additional elements are required for its operation as a computer: display, mouse, keyboard, SD memory, and power supply. Connection to the network can be made via Wi-Fi or directly via ethernet cable connection.

### 2.2. Components

Before deciding the type of components that the instrumented e-scooter should include, the variables to be measured were defined. In this regard, the main goal of the e-scooter is to assess the behaviour, safety, and comfort of users riding the e-scooter and the operation of the micromobility users interacting with them. As mentioned above, the main variables to be collected are the speed, accelerations, and location of the e-scooter as well as the speed and lateral clearance left by the micromobility user overtaking or meeting the e-scooter.

Based on these variables, the electronic devices to be included in the e-scooter were selected according to the availability of the element, cost-performance ratio, size, and compatibility with the Raspberry Pi 4B. All components are described in Table 1.

Two ultrasonic sensors HC-SR04 were used to measure lateral clearance from the front and rear part of the instrumented e-scooter to other micromobility users and to the physical barriers of the infrastructure. This type of device has a distance measurement range from 4 cm to 450 cm. Its operation consists of sending a pulse of 8 cycles of 40 kHz after receiving a trigger signal of at least 10 μs. If it detects an echo in the cycle, the sensor sends back a pulse with a duration in μs proportional to the time it takes to receive the echo signal. Knowing the time it takes to receive the signal and the speed of sound, the distance at which an object is located is obtained.

These sensors need a power supply of 5 V, returning this voltage to the Raspberry Pi which could damage the GPIO pin. To avoid malfunctions, an electric circuit is designed with resistances that help reduce the voltage. Then, a 2 kΩ resistor was placed between the Ground and Echo pins as a protection in case of a programming error, so the current is sent to Ground instead of Echo pin. Moreover, the Raspberry must receive 3.3 V from the sensors. Therefore, with previous 2 kΩ resistor and applying Equation (1), an additional 1 kΩ resistor had to be placed.

(1) $$V_{out}=V_{in}\frac{R_{2}}{R_{1}+R_{2}}\;V_{out}=V_{in}\frac{R_{2}}{R_{1}+R_{2}}\;3.3\;V=5\;V\frac{2000\;\Omega }{R_{1}+2000\;\Omega }\;3.3\;V*\left(R_{1}+2000\right)\;\Omega =5\;V*2000\;\Omega \;R_{1}=\frac{10,000\;A-6600\;A}{3.3\;V}=1030\;\Omega$$

The component responsible of calculating vertical accelerations was the Pololu MinIMU-9 v5. It is a compact (20.32 mm × 12.70 mm) board that combines ST’s LSM6DS33 3-axis gyroscope and 3-axis accelerometer and LIS3MDL 3-axis magnetometer to form an Inertial Measurement Unit (IMU). The nine independent rotation, acceleration, and magnetic readings provide all the data needed to make an attitude and heading reference system (AHRS).

The Garmin VIRB Elite video camera was selected because the authors have some of them and they were previously used for bicycle instrumentation [4]. The video camera allows diverse video modes, including high-definition recording at 60 fps. Additionally, the device records a continuous track log for each video recording with a frequency of 10 Hz. The video camera is used to measure the speed of the instrumented vehicle, identify the interactions with other micromobility users, and record the location of the instrumented e-scooter.

In addition to previous components, the system is completed with a Green Cell PowerPlay external battery with a 20,000 mAh nominal capacity allowing data collection for 12 h, an SD card for data storage, and metal structures for the installations of the diverse sensors.

Summarizing, the e-scooter accelerations are collected by the IMU, lateral clearance during meeting and overtaking manoeuvres is gathered by the ultrasonic sensors, and e-scooter speed and position are obtained from the Garmin VIRB Elite video camera. In addition, the speed of the micromobility user meeting or overtaking the e-scooter can be estimated from the relative speed obtained from the ultrasonic sensors. The cost of the complete kit consisting of high-quality sensors would be ~4000 euros, while the developed DAS costs ten times less (~395 euros, including assembly).

### 2.3. Software

**Figure 2:**
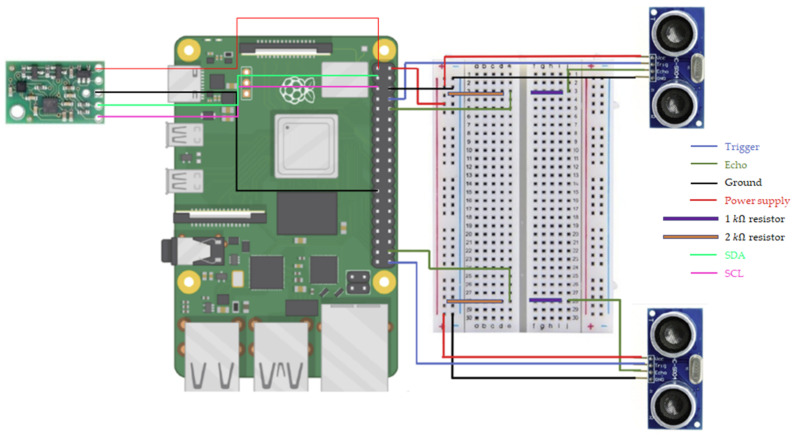
Connection of electronic devices to the Raspberry Pi 4B.

The Raspberry Pi 4B has 4 USB ports, 2 micro HDMI connections, an ethernet connection port, a pi camera connection, a power connection, and 24 GPIO pins. The GPIO pins are the most important part as they connect the sensors with which the data acquisition will be performed. Figure 2 shows the connections of the IMU and ultrasonic sensors to the Raspberry Pi 4B. Given that the Raspberry Pi has two 5V supply pins, each ultrasonic sensor was connected to each of them. In addition, the IMU needs to be connected to the Raspberry Pi through 4 pins: supply (at 3.3 V), Ground, and the SDA and SCL GPIOs. These pins allow data exchange and determine the frequency of the readings without any intermediate element.

The motherboard runs Linux Raspbian, installed from a MicroSD card. It includes necessary software for many areas of work (e.g., Internet browser, programming tools, or Open Office). The most relevant software is Thonny Python 3, a Python IDE used to program all sensors connected to the Raspberry Pi. The sensors were programmed on Python 3.7, using open-source libraries.

The IMU needs to be calibrated before being used. First, the gyroscope calibration consisted of taking 10,000 readings for the three axes while the IMU is stationary and calculating the average deviation of each axis from 0. As a result, the average deviations for *x*-axis, *y*-axis, and *z*-axis were 0.04134, −0.11321, and −0.03938, respectively. These values will be then subtracted from the raw data gathered. Secondly, the accelerometer was calibrated. This sensor was placed in different positions so that every axis gathered data with the axis pointing up, down, and perpendicular to the action of gravity, since these directions are the known theoretical values (1g, −1g and 0). Once the raw data in those positions were gathered, the calibration consisted of fitting Equation (2).

(2) $$y=m_{x}\cdot x+b\;,$$

where y is the theoretical value, x is the gathered data, $m_{x}$ is the slope, and b is the intercept. Table 2 summarizes the values of $m_{x}$ and *b* for each axis.

Applying the calibrated equations to the data collected directly by the sensors will convert the raw data into the calibrated data.

Data registered by the devices—IMU and ultrasonic sensors and video camera—were stored in different files. Data gathered from the ultrasonic sensors (timestamp and lateral clearance) and the IMU (timestamp and vertical accelerations) were saved in the Raspberry Pi as CSV files. For that purpose, two independent scripts programmed in Python version 3.7 were developed.

**Figure 3:**
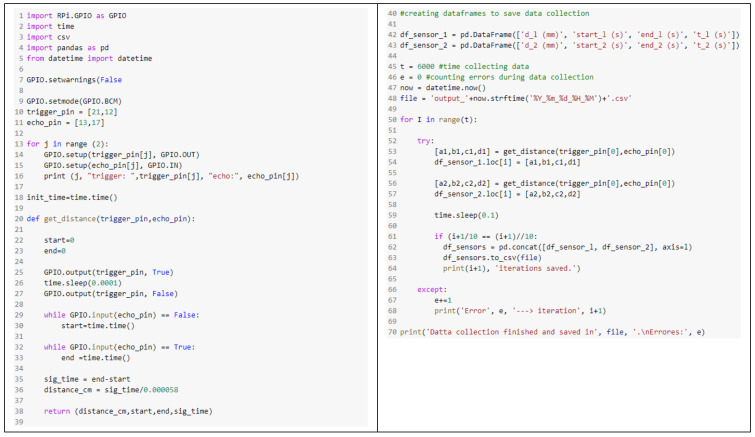
Script programmed in Python for the use of ultrasonic sensor HC-SR04.

Regarding the code associated with the ultrasonic sensors, the first step is to import the *GPIO*, *time*, and *pandas* libraries whose functions are to activate the GPIO pins on the board, obtain time measurements and create and write the output file, respectively (Figure 3, lines 1–5). Secondly, the GPIO pins are programmed as output for Trigger and input for Echo. To do this, the number of pins in which each of the sensors is connected to the Raspberry Pi must be provided (Figure 3, lines 9–16). Then, the time counting starts. Next, the function with which the sensors will be triggered to take the distance measurements is created (Figure 3, lines 20–38). This function returns the distance, the initial and final time of the Echo pin measurement, and the difference between these timestamps. Afterwards, the DataFrames that store the data are created (Figure 3, lines 42–44). Subsequently, a loop is programmed with which the sensors take measurements at the same time and the data are saved. With the programming, it has been established that the pulses are sent with a constant time interval, guaranteeing a minimum rest time of 100 ms between one trigger and another. Finally, in the event of an error during execution, the code will send an error message.

**Figure 4:**
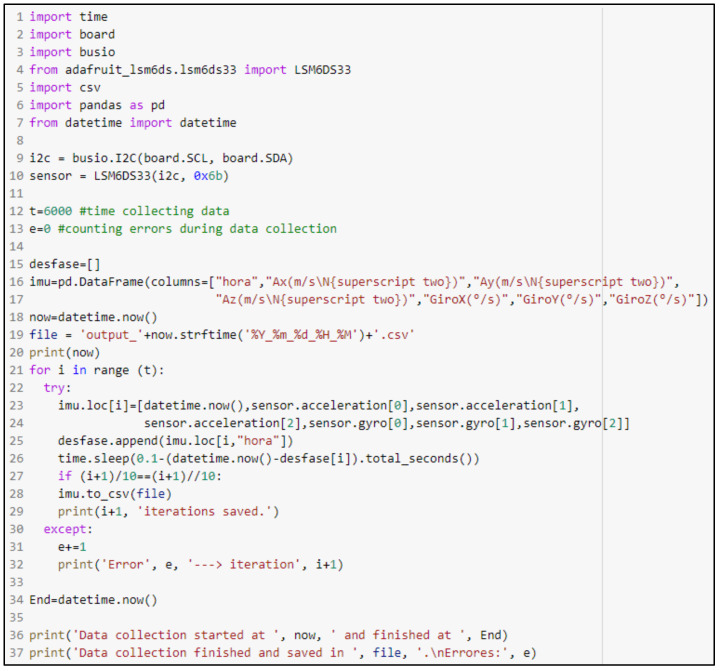
Script programmed in Python for the use of MinIMU-9 v5.

The script for the MinIMU-9 v5 is included in Figure 4. First, the libraries needed to process and extract the data from the IMU are imported (Figure 4, lines 1–7). To this regard, *busio* and *board* libraries are used to define the sensor connection to the Raspberry Pi, while *adafruit_lsm6ds.lsm6ds33* library is needed to extract the data from the IMU. The next step is to indicate the position of the IMU on the Raspberry Pi motherboard (Figure 4, lines 9–10). Next, the DataFrame that stores the data collected by the IMU is created. Then, a loop is programmed to measure the accelerations, saving the data every 10 readings (Figure 4, lines 21–32). Although the frequency of data collection was set at 10 Hz, it could be adjusted to obtain data with higher frequency (Figure 4, line 26). In addition, to synchronize the video recorded by the Garmin VIRB Elite video camera and the IMU data, the code prints the start and end time of data collection in Central European Time (CET) format (Figure 4, line 36). As in the case of the ultrasonic sensors, error checking during execution has also been included in the code.

For data collection, VNC Connect software and VNC Viewer app were installed on the Raspberry Pi and on a smartphone, respectively. This software allows remote control of the sensors from the smartphone which is connected via USB to the Raspberry Pi.

In addition, the video camera stored the video recording in an MP4 file and the georeferenced data in a GPX file. While the CSV files on the Raspberry Pi are named with the start time and date of each run, the video camera files follow the correlative naming encoding of the camera, but both are named the same (e.g., VID_001.MP4 and VID_001.gpx).

### 2.4. System Design

Once the devices were checked for proper and accurate operation, they were assembled on the e-scooter. Regarding this, the location of the devices was carefully studied so as to minimise their influence on the behaviour of the riders, ensure sufficient space for a proper operation of the vehicle, and guarantee the safety of the user, using lightweight materials and maintaining the stability of the vehicle.

The Garmin VIRB Elite video camera was placed on the front of the vehicle, specifically on the handlebar of the e-scooter by means of a camera mount, because the video recording was also used to identify interactions with other micromobility users.

The system was also conceived to register vertical accelerations caused by the vibrations during riding, which can be used as a surrogate measure of riding comfort. For this purpose, the IMU was placed close to the gravity centre of the e-scooter to ensure a more realistic reading of the vibrations experienced by micromobility users.

**Figure 5:**
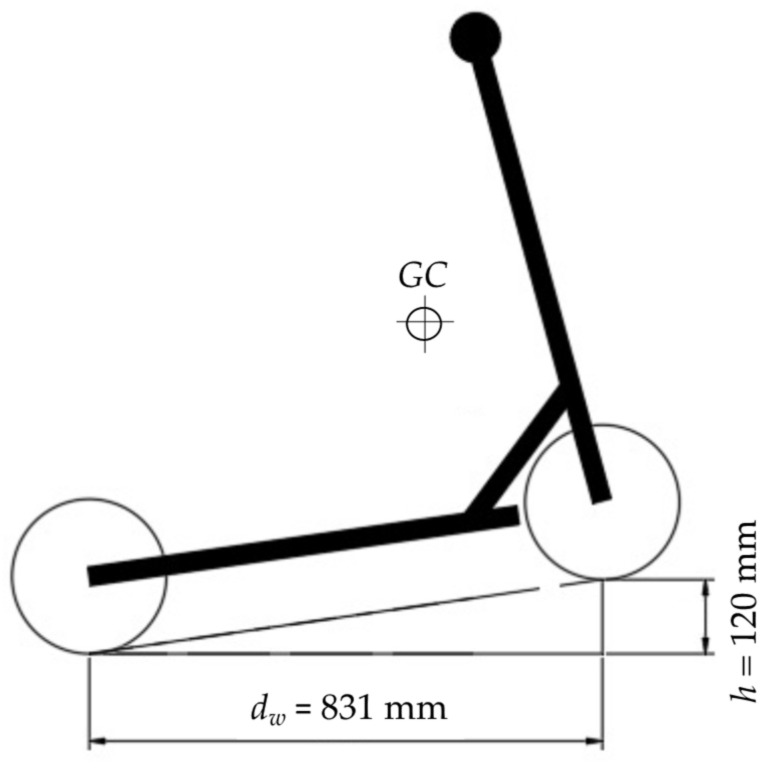
Centre of gravity of the e-scooter.

To find the centre of gravity (*GC*), the e-scooter was weighted while the rear wheels were on the ground. Then, knowing the distance between wheels’ axes (*d_w_*), the height difference between both of them (*h*), the measured weight, and considering the specific weight and height of a rider (~70 kg and ~1.70 m), the *x*-axis position of the gravity centre was around 20 cm from the front wheel and the *y*-axis position around 50 cm above the deck of the e-scooter (Figure 5).

**Figure 6:**
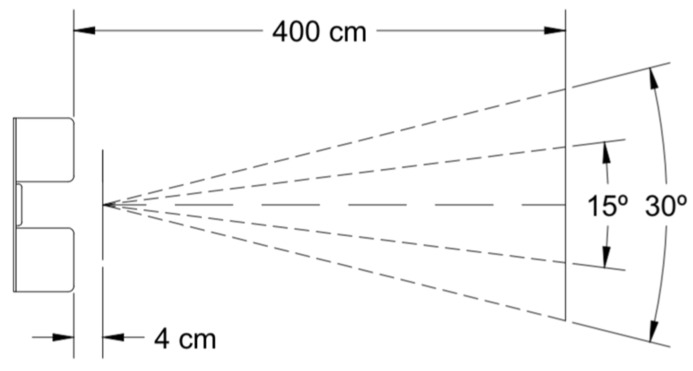
Range of the ultrasonic sensor.

**Figure 7:**
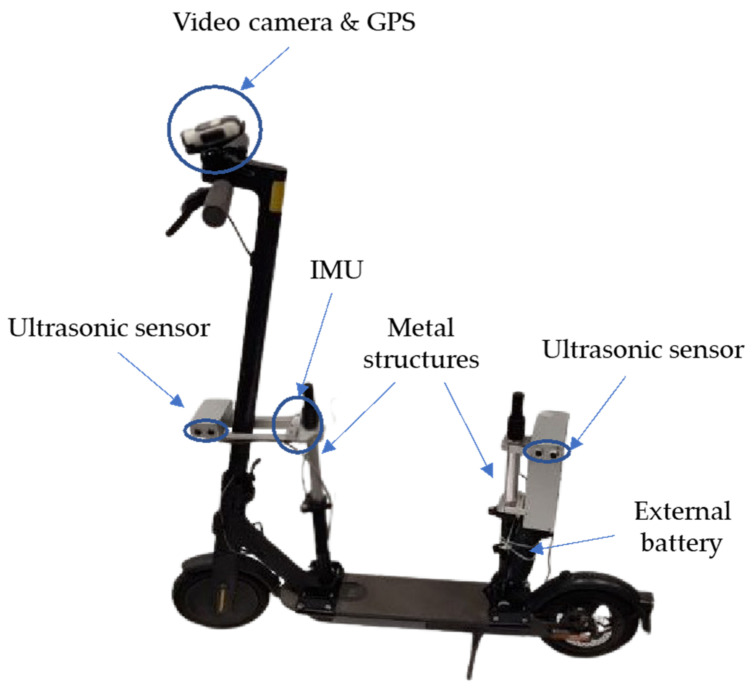
Instrumented e-scooter.

In addition, the ultrasonic sensors are responsible for collecting lateral clearance during overtaking and meeting manoeuvres. In this way, these sensors are required to be facing the left side of the vehicle, since both type of manoeuvres occur on that side. They must also be placed at a sufficient height so that the sound does not bounce off the pavement, preventing malfunction during data collection. Since the aperture angle is 15°, the ultrasonic sensors should be located at least at 50 cm above the ground (Figure 6). Therefore, two support structures were designed and installed at the front and rear of the e-scooter to mount these sensors (Figure 7).

The structure placed at the front of the e-scooter required an extra element to make sure the sensors are fixed in front of the vehicle, not being affected by vibrations and turns of the handlebar. This piece also allocated the IMU since it was close to the GC. The back structure was complemented with a greater metallic box that contained, in addition to the ultrasonic sensor, the Raspberry Pi (Figure 7). Both structures are rigidly fastened to the e-scooter, while the sensors are rigidly attached to the structures.

Only 4 cables go out of the back box: (i) connection for the ultrasonic sensors placed at the front, (ii) connection for the IMU, (iii) power supply, and (iv) Internet supply. The connection cables for the front sensors are attached to the underside of the deck of the e-scooter and to the front structure so that they do not become entangled with feet or external elements. As for the power supply cable, since the battery is placed just below the box, there is no need for a long cable. The last cable is used to connect the Raspberry Pi to the rider’s smartphone, from which Internet will be supplied to the motherboard. This allows the rider to remotely control the Raspberry Pi. The smartphone can be placed on the handlebars using a holder or stored in the rider’s pocket. Given that the camera is not connected to the Raspberry Pi, the only way to synchronize the data logs from both devices and the video camera is based on the recording start time of both systems which are available in Central European Time (CET) format. Data filtering uses the recording start time of both systems—sensors and video cameras—to remove data recorded only by one system. This procedure ensures a synchronization accuracy of 0.01 s.

Figure 7 shows the fully equipped e-scooter.

The instrumented e-scooter was tested to evaluate its applicability for the analysis of the lateral clearance during meeting and overtaking manoeuvres and the study of riding comfort. First, the ultrasonic sensors were validated by comparing the distance provided by these sensors with the actual distance to objects at known distances, in static and moving conditions. Distances of 125, 250, 500, 1000, 1500, 2000, and 2500 mm were considered for the test because the lateral clearance between micromobility users is usually less than 2 m, and the width of the bike lane rarely exceeds 2.5 m. As a result, a root mean square error of 17 mm was identified, validating the use of these devices.

**Figure 8:**
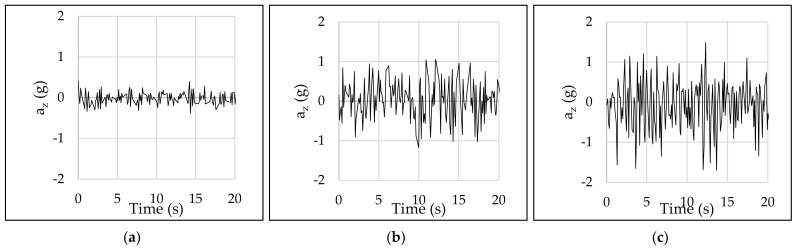
IMU test: (**a**) asphalt pavement, (**b**) concrete pavement, and (**c**) cobble pavement.

The IMU was tested riding the e-scooter at 20 km/h along diverse types of pavements with different surface roughness—asphalt, concrete, and cobble pavements. Given that the main goal is measuring users’ riding comfort, the absolute value of vertical acceleration is not as important as the differences in acceleration between different types of pavements. Specifically, the vertical acceleration ranged between ±0.5 g, ±1.0 g, and ±1.5 g for asphalt, concrete, and cobble pavements, respectively (Figure 8). This fact reveals that there are significant differences in terms of vertical accelerations among the studied pavements and, therefore, it is possible the proposal of surrogate measures for riding comfort, validating the usefulness of the IMU device.

## 3. Case Study

**Figure 9:**
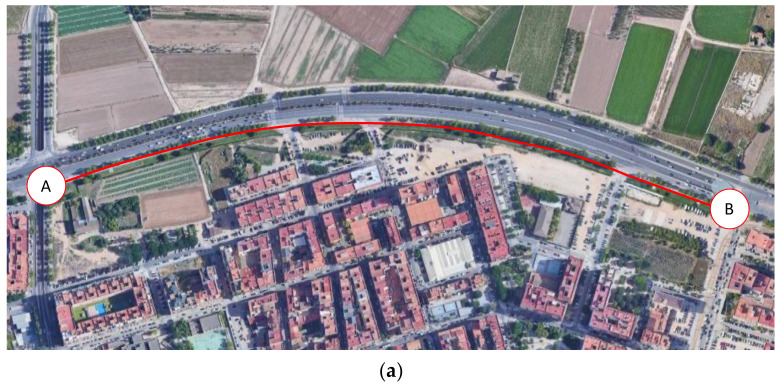

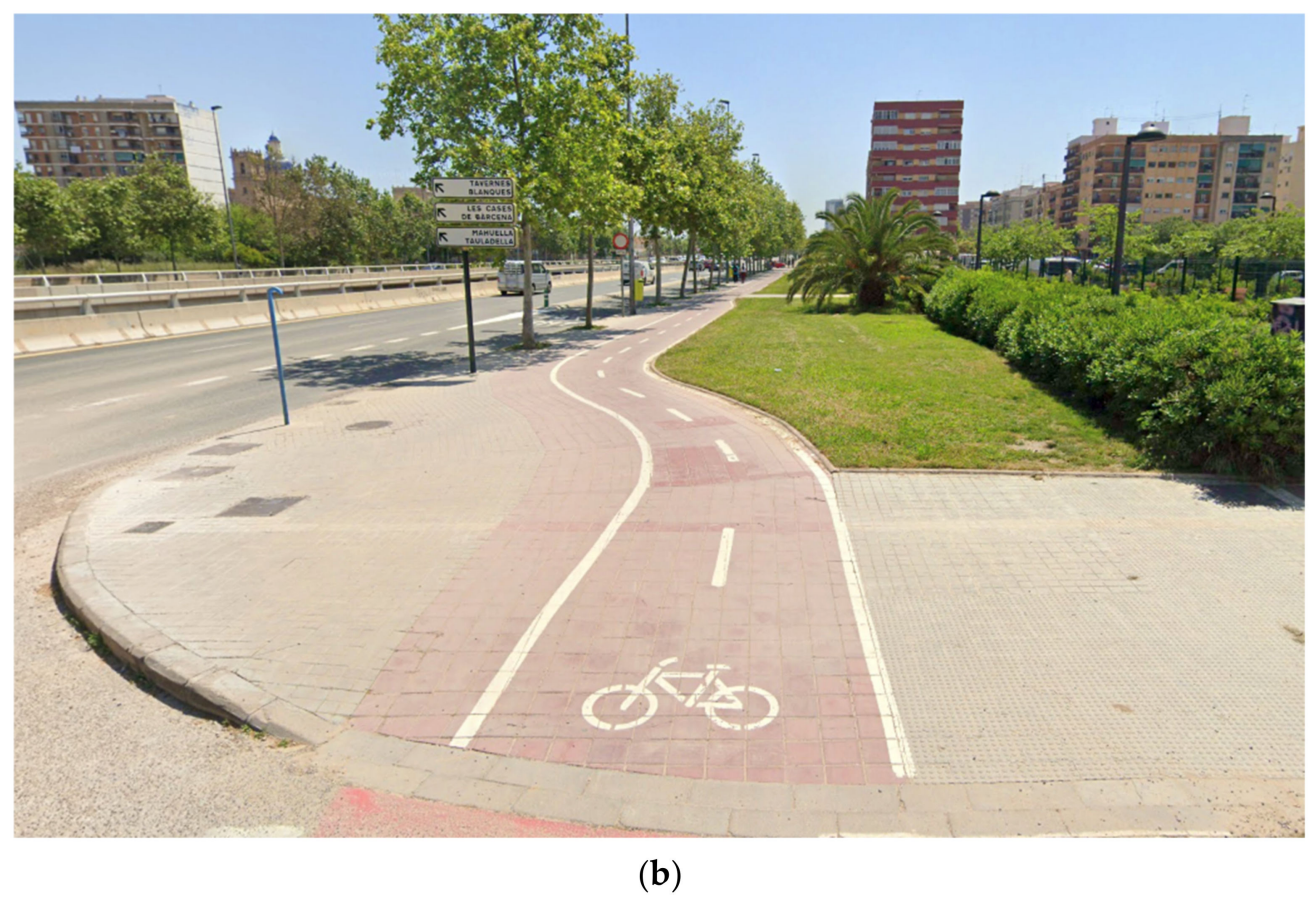
Case study location: (**a**) aerial view (A: start point; B: final point) and (**b**) bike lane configuration.

Once the e-scooter was equipped, a case study was developed along a side path—an off-street bikeway built as an extension of the sidewalk—located in the city of Valencia (Spain) (Figure 9). The length and width of the selected segment were 850 m and 2 m, respectively. Moreover, the pavement of the bike lane consisted of painted tiles.

Table 3 includes some data registered by the IMU that include the time and acceleration (*a*) and angular speed (*w*) for every axis. Taking into account the road segment is straight, the e-scooter is stopped at the beginning of data collection, and the location of the IMU, the raw data makes sense. In this regard, it should be noted that *x*-axis acceleration is associated with transverse acceleration and *y*-axis acceleration is associated with longitudinal acceleration. As can be observed, the longitudinal acceleration (*a_y_*) is positive because the rider is speeding up whereas the transverse acceleration (*a_x_*) and angular speeds are around 0 m/s^2^ because the rider is describing a straight path with almost no transverse movements. Moreover, the vertical acceleration (*a_z_*) registered the vibration of the vehicle that can be used to study the comfort of micromobility users. The values of *a_z_* are around the acceleration of the gravity while pavement evenness is adequate, but this variable increases as the pavement condition worsens or with the presence of pavement distresses.

**Figure 10:**
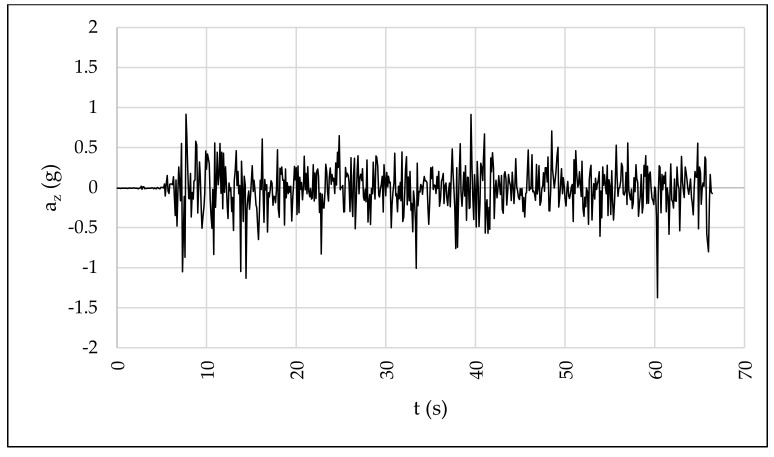
Vertical accelerations during data collection.

Due to the importance of the vertical acceleration, Figure 10 shows *a_z_* over time after the corresponding correction of *a_z_* with the parameters included in Table 2. This plot can be used to identify specific pavement distresses that are associated with peak values. In addition, diverse surrogate measures can be defined from these data to assess riding comfort along bike lanes.

**Figure 11:**
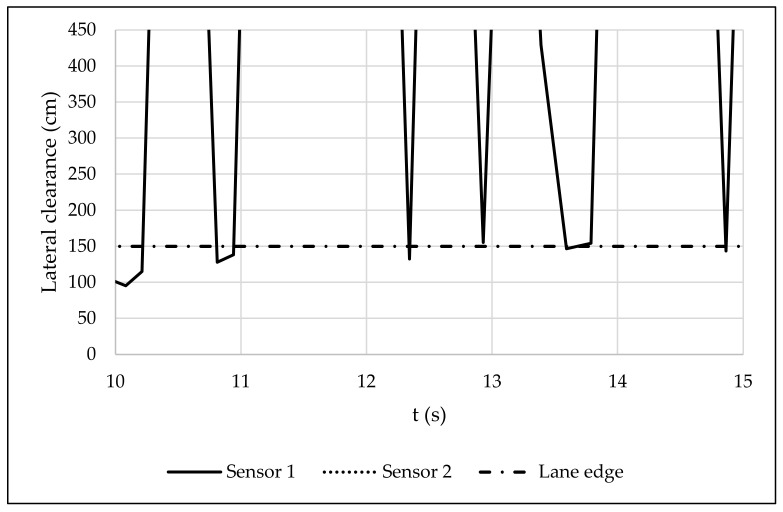
Lateral clearance during data collection.

A sample of the data gathered through the ultrasonic sensors is summarized in Table 4. For each ultrasonic sensor, the lateral clearance (*d_i*), the time when the sound is emitted (*start_i*), the time when the sound is received (*end_i*), and the time-lapse between emission–reception (*t_i*) are shown. Figure 11 plots lateral clearance over time, with “lane edge” being the distance between the path of the rider travelling in the centre of the lane and the edge of the side path. In this regard, an overtaking or meeting manoeuvre is associated with sudden reductions in lateral clearance.

**Figure 12:**
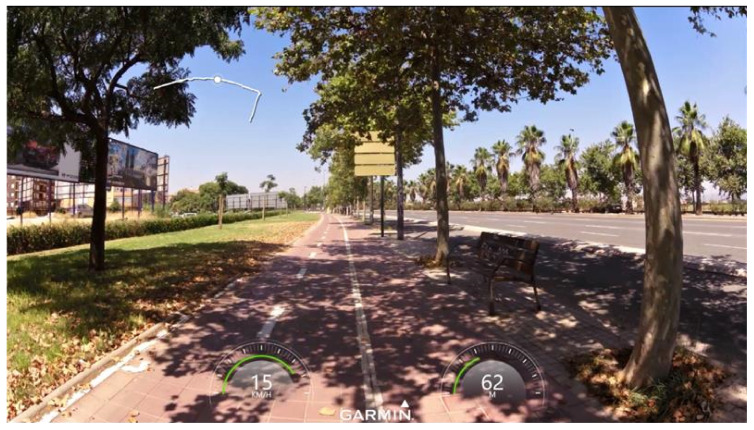
Screenshot from VIRB Edit software.

Figure 12 shows a screenshot extracted from the VIRB Edit software of the video recording. The Garmin VIRB Elite video camera was set to 1080p mode, recording at a resolution of 1920 × 1080 pixels and at 30 fps. The VIRB Edit software links speed, position, and altitude data, displaying them all together when viewing the video recording (Figure 12). Additionally, the video recording can be used to identify the interactions with other micromobility users. In fact, the data obtained from the video camera and from the sensors were synchronized to distinguish between meeting and overtaking manoeuvres. Specifically, more information about the use of the instrumented e-scooter for the analysis of micromobility users’ behaviour during meeting manoeuvres can be found in Fonseca-Cabrera et al. [20].

To conclude the case study, it should be highlighted that the e-scooter equipped by a low-cost data acquisition system is able to provide accurate and reliable data to study and assess the operation of micromobility users. While the IMU collects acceleration data that can be useful to assess the comfort of micromobility users, the ultrasonic sensors can be used to determine lateral clearance and thus characterize overtaking and meeting manoeuvres. Last but not least, the Garmin VIRB Elite video camera provides speed and position data and allows identifying interactions with other micromobility users.

## 4. Discussion

The main variables used to characterize the behaviour, safety, and comfort of a micromobility user are speed and accelerations, lateral clearance during overtaking and meeting manoeuvres, speed of interacting users, and the vibrations experienced when riding along cycling facilities.

In order to collect this data, many studies proposed the instrumentation of a bicycle with several devices, such as GPS, cameras, distance sensors, speedometers, and gyroscopes [4]. However, these devices are not usually implemented in the same system and are very expensive. Therefore, recent studies have developed their own custom-made devices for data collection based on Arduino and Raspberry Pi, among others [5,6,7,8].

However, in recent years, the use of other personal mobility vehicles, such as e-scooters, has considerably increased. Since there is not a specific infrastructure for these new users, they ride along cycling facilities interacting with bicyclists. Thus, the analysis of the behaviour of these new vehicles and the interactions among them and with bicycles is of great interest. In addition, since bike lanes are designed without considering other micromobility users with different characteristics, it is also worth studying the vibrations experienced by the users of e-scooter.

Recent studies have focused on this last area of study. Some of them have collected the necessary data with a smartphone application [17,18]. The main drawback is that the accuracy and frequency of the sensors may vary among different smartphones. Other studies have used sensors based on Raspberry Pi or Arduino installed on an e-scooter. Cano-Moreno et al. [19] used accelerometers with a data collection frequency of 200 Hz, which is too high and storage-consuming. Ma et al. [16] used an inertial measurement unit (IMU) combining a three-axis gyroscope and three-axis accelerometer, being consistent with the system proposed in this study. In this regard, the proposed system includes an IMU close to the gravity centre of the e-scooter to ensure a more realistic reading of the vibrations experienced by micromobility users.

Ma et al. [16] also included a LIDAR Scanner to identify obstacles and elements close to the e-scooter and the distance to them. However, they did not consider specifically the possibility to measure the distance between users meeting or passing each other. A LIDAR can be used for this purpose, but the data storage and processing effort is too high. Therefore, the system proposed in this study includes two ultrasonic sensors to measure lateral clearance from the front and rear parts of the instrumented e-scooter to other micromobility users and to the physical barriers of the infrastructure. Their range can vary from 2 cm to 450 cm, allowing to set it depending on the aim of the research. Moreover, their high frequency of data collection (40 kHz) allows for registering lateral clearance on both distance sensors during the meeting and overtaking manoeuvres and, additionally, the speed of passing vehicles. This frequency is even higher than the frequency of the devices of instrumented bicycles used for lateral clearance detection during overtaking manoeuvres by motorized vehicles travelling at high speeds [5,6,7]. Nevertheless, during some meeting manoeuvres, only one of the sensors collected data, so the system could be improved including sensors with high frequency when studying this type of manoeuvres.

Regarding the proposed video camera, the Garmin VIRB Elite video camera has been used because it was available at the research group and integrated a GPS unit. However, similar video cameras, such as the one used by Henao et al. [8], could be installed. In that case, an external GPS device could be needed. Regarding this, GPS data can be used to track the e-scooter and estimate its speed. However, since in urban environments it is common to lose the GPS signal, it is suggested to develop data collection at a steady speed to minimise the influence of this factor on the analysed phenomena—micromobility users’ interactions or vertical acceleration. Regarding this, the travel speed is known since the e-scooter displays the speed in real time while riding.

The use of these sensors allows for gathering the data needed for micromobility safety research with a smaller size and lower price than other high-quality sensors, but with an adequate data range and accuracy. A comparison between the specifications of the sensors used for the instrumented e-scooter and those of other high-quality sensors is shown in Table 5. Moreover, the cost of the developed DAS (~€395) is around ten times lower than the cost of the complete kit consisting of high-quality sensors (~€4000).

The data acquisition system can be easily complemented with other sensors to expand its use. For instance, the implementation of two distance sensors placed in the front and in the rear part of the e-scooter would allow the analysis of the safety distance with preceding and following micromobility users. In addition, a device for the assessment of the user’s subjective risk during passing or meeting manoeuvres could be installed on the handlebar.

Considering the data gathered by the instrumented e-scooter, it can be used not only for research purposes but also for cycling facility inspections. In the case of its use for research, the designed instrumented e-scooter can be the basis of naturalistic data collection methodology in studies focusing on: (i) road safety analysis of micromobility based on surrogate measures such as speed, accelerations, and lateral clearance, (ii) analysis of riding comfort, comparing vertical accelerations with micromobility users perceptions, (iii) improvement of the methodology for estimating the level of service of cycling facilities, considering both bicycles and e-scooters. Moreover, road administrations and operators can use this instrumented e-scooter as a low-cost mobile sensor to carry out a variety of studies, including: (i) road safety inspections through video recordings, allowing the identification of points with poor visibility, poor maintenance of signage, and pavement condition; (ii) pavement deterioration assessment through the analysis of vertical accelerations over time, improving decision-making in pavement maintenance and management; (iii) identification of pavement distresses through processing image techniques based on artificial intelligent, achieving an automatic inventory of pavement damages; and (iv) cycling demand studies through the estimation of the intensity and speed of micromobility users by using data from video recordings and ultrasonic sensors.

Although the proposed data acquisition system guarantees promising results for research and engineering purposes focused on micromobility, some limitations have been identified. A full charge in the e-scooter battery allows travelling up to 30 km, which could make data collection difficult in cities with long bike lane networks. However, an external battery for the e-scooter could be added to the system. On the other hand, given that the video camera is not automatically synchronised with the devices connected to the Raspberry Pi, a manual adjustment is needed beforehand to synchronise GPS and speed data with vibrations and lateral clearance data. Additionally, difficulties in estimating the speed of other users interacting with the instrumented e-scooter were detected. This fact was associated with the characteristics of micromobility vehicles and the location of ultrasonic sensors. Although the micromobility users interacting with the instrumented e-scooter were properly detected by the ultrasonic sensors, the location of the collision of the sound wave with the vehicles, which has a significant impact on the speed estimation, is unknown. This could be solved by installing 2D laser devices, but the cost of the instrumentation would be considerably higher.

Finally, it should be noted that the data acquisition system has been designed to be used for any type of bike lane, including shared lanes where micromobility users interact with motorized vehicles.

## 5. Conclusions

The rise in the popularity of e-scooters is changing mobility patterns and lifestyles worldwide. This type of vehicle is the second most widely used for micromobility, after bicycles. However, although there are lots of studies on the safety and behaviour of cyclists, the number of studies on e-scooter users is scarce. Moreover, although they share the same facilities, their characteristics are different, so the results obtained for cyclists cannot be directly applied to e-scooters. Therefore, further research is needed.

This study proposes a data acquisition system that has been implemented on an e-scooter to be used for naturalistic data collection in micromobility-related research. The main requirements for the definition of this system were cost and size. Thus, a low-cost open-source data acquisition system based on Raspberry Pi has been developed. The use of Raspberry Pi technology has allowed the development of a system for less than 400€ and with a size compatible with the installation on an e-scooter.

The instrumented e-scooter is able to gather the necessary data to characterize the position, speed, and accelerations of the e-scooter and the speed and lateral clearance left during overtaking and meeting manoeuvres. To this regard, the instrumentalization consists of two ultrasonic sensors HC-SR04 located at the front and rear of the e-scooter and an inertial measurement unit Pololu MinIMU-9 v5 located at the gravity centre of the user-vehicle system, both connected to a single board computer (Raspberry Pi 4B). In addition to these devices, a Garmin VIRB Elite video camera with a GPS unit was installed on the handlebar of the e-scooter. The system is completed with an external battery, an SD card, and some metal cases for the installation of the sensors.

Once the e-scooter was fully instrumented, it was subject to different tests in controlled laboratory conditions to assess its suitability for the study of road safety, behaviour, and comfort of micromobility users. In addition, a case study was carried out on a bike line segment. The results show that the instrumented e-scooter can be used for the abovementioned purpose and is robust enough to be used for naturalistic data collection, with the only limitation being the battery life of the e-scooter.

As a conclusion, a low-cost open-source data acquisition system based on Raspberry Pi has been developed and implemented on a e-scooter. The proposed system allows highway engineers and urban mobility planners to collect data to analyse the behaviour, safety, and comfort of e-scooter users. Additionally, thanks to the small size and the adaptability of the sensors, the system can be installed not only on an e-scooter but also on a bicycle or other personal mobility vehicles.

Finally, it should be noted that this instrumented e-scooter can be used for research purposes but also to assess pavement condition and riding comfort of micromobility users in a cycling network when the budget is limited.

## Figures and Tables

**Table 1:** Summary of components.

Device	Model	Manufacturer	Specifications	Quantity	Price (€)
IMU	MinIMU-9 v5	Pololu (Las Vegas, NV, USA)	Axes: pitch (x), roll (y), yaw (z) Gyro range (º/s): ±125, ±245, ±500, ±1000, ±2000 Accelerometer range (g): ±2, ±4, ±8, ±16 Magnetometer range (gauss): ±4, ±8, ±12, ±16 Voltage: from 2.5 V to 5.5 V Frequency: 400 kHz	1	~20
Ultrasonic Sensor	HC-SR04	Naylamp Mechatronics (Trujillo, Peru)	Range: 2–450 cm Frequency: 40 kHz Accuracy: ±3 cm Voltage: 5 V	2	~5
Video camera GPS	Garmin VIRB Elite	Garmin (Lanexa, KS, USA)	Camera resolution: HD1080p Camera’s field of view: 120° Sensor: 1/2.3-inch CMOS with 16 MP	1	~250
			GPS frequency: 10 Hz		
Single board computer	Raspberry Pi 4 Model B	Raspberry Pi Foundation (Cambridge, UK)	Connectivity: Bluetooth, Wi-Fi, Ethernet, USB 2.0 and USB 3.0 Voltage: 5 V Processor: Broadcom BCM2711, Quad core Cortex-A72 (ARM v8) 64-bit SoC @ 1.5 GHz	1	~70
Battery	Green Cell PowerPlay^20^	CSG S.A. (Cracow, Poland)	Nominal Capacity: 20,000 mAh Output: 5–9 V/2 A, 12 V/1.5 A Input: 5–9 V/2 A	1	~38
SD Card	SanDisk Ultra	SanDisk Corporation (Milpitas, CA, USA)	Storage capacity: 32 GB	1	~12
Seat Structures				2	~120
				TOTAL	~395

**Table 2:** Calibration of accelerometer.

Axis	$m_{x}$	*b*
x	0.10263603	−0.00906621
y	0.10225977	−0.00367127
z	0.10153481	−0.03342624

**Table 3:** IMU data output.

	Time	*a_x_* (m/s^2^)	*a_y_* (m/s^2^)	*a_z_* (m/s^2^)	*w_x_* (°/s)	*w_y_* (°/s)	*w_z_* (°/s)
0	11:30.4	−0.571	0.100	−10.881	−0.002	−0.178	−0.170
1	11:30.5	1.017	1.273	−7.513	0.157	−0.054	−0.282
2	11:30.6	−1.560	2.243	−7.238	−0.008	−0.141	−0.141
3	11:30.7	−1.685	4.443	−8.088	0.016	−0.246	−0.044

**Table 4:** Ultrasonic sensor data output.

	*d_1* (mm)	*start_1* (s)	*end_1* (s)	*t_1* (s)	*d_2* (mm)	*start_2* (s)	*end_2* (s)	*t_2* (mm)
0	1505.830	0.000	0.070	0.070	592.039	0.097	0.132	0.034
1	1505.777	0.240	0.310	0.070	1506.776	0.323	0.393	0.070
2	1505.662	0.501	0.571	0.070	1506.752	0.582	0.652	0.070
3	1505.662	0.761	0.831	0.070	1506.616	0.839	0.909	0.070

**Table 5:** Sensor specifications comparison.

Device	Model	Manufacturer	Specifications
IMU	MinIMU-9 v5	Pololu (Las Vegas, NV, USA)	Gyro range (º/s): ±125, ±245, ±500, ±1000, ±2000 Accelerometer range (g): ±2, ±4, ±8, ±16 Frequency: 400 kHz
	RLVBIMU04	Racelogic (Buckinham, UK)	Gyro range (º/s): ±450 Accelerometer range (g): ±20 Frequency: 100 kHz
Distance sensor	HC-SR04	Naylamp Mechatronics (Trujillo, Peru)	Range: 2–450 cm Frequency: 40 kHz Accuracy: ±3 cm
	TruSense S200	Laser Technology, Inc. (Centennial, CO, USA)	Range: 46–750 cm Frequency: 200 kHz Accuracy: ±4 cm
GPS	GPS of Garmin VIRB Elite camera	Garmin (Lanexa, KS, USA)	GPS frequency: 10 Hz
	GPS of Video VBOX	Racelogic (Buckinham, UK)	GPS frequency: 10 Hz

## Data Availability

The data presented in this study are available on request from the corresponding author. The data are not publicly available due to a non-disclosure agreement.
